# Description and phylogenetic analysis of the complete mitochondrial genome in *Eulaelaps silvestris* provides new insights into the molecular classification of the family Haemogamasidae

**DOI:** 10.1017/S0031182023000616

**Published:** 2023-08

**Authors:** Hui-Juan Yang, Zhi-Hua Yang, Tian-Guang Ren, Wen-Ge Dong

**Affiliations:** 1 Institute of Pathogens and Vectors, Yunnan Provincial Key Laboratory for Zoonosis Control and Prevention, Dali University, Dali, Yunnan 671000, China; 2 School of Foreign Languages, Dali University, Dali 671000, China; 3 College of Nursing, Dali University, Dali 671000, China

**Keywords:** *Eulaelaps silvestris*, Haemogamasidae, mitochondrial genome, phylogeny

## Abstract

In this study, the mitochondrial genome of *Eulaelaps silvestris*, which parasitizes *Apodemus chevrieri*, was sequenced and assembled to fill the gap in understanding the molecular evolution of the genus *Eulaelaps*. The *E. silvestris* mitochondrial genome is a double-stranded DNA molecule with a length of 14 882 bp, with a distinct AT preference for base composition and a notably higher AT content than GC content. The arrangement between genes is relatively compact, with a total of 10 gene intergenic regions and 12 gene overlap regions. All protein-coding genes had a typical ATN initiation codon, and only 2 protein-coding genes had an incomplete termination codon T. Out of the 13 protein-coding genes, the 5 most frequently used codons ended in A/U, with only 1 codon ending in G/C had an relative synonymous codon usage value >1. Except for *trnS_1_* and *trnS_2_*, which lacked the D arm, all other tRNAs were able to form a typical cloverleaf structure; and there were a total of 38 mismatches in the folding process of tRNA genes. Unlike the gene arrangement order of the arthropod hypothetical ancestor, the *E. silvestris* mitochondrial genome underwent fewer rearrangements, mainly near tRNA genes and control regions. Both the maximum likelihood tree and the Bayesian tree showed that the family Haemogamasidae is most closely related to the family Dermanyssidae. The results not only provide a theoretical basis for studying the phylogenetic relationships of the genus *Eulaelaps*, but also provide molecular evidence that the family Haemogamasidae does not belong to the subfamily Laelapidae.

## Graphical abstract

**Figure:**
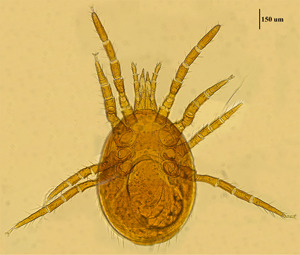

## Introduction

Mites are incredibly diverse and abundant in the world, with an estimated total species count between 500 000 and 1 000 000, although only around 55 000 species have been described (Manu *et al*., 2021). Mites have a complex habitat and a wide range of feeding activities, acting as predators, phytophagous, decomposers and fungivores to scavengers or parasitic on other animals (Badieritakis *et al*., 2014). Currently, some Mesostigmata species have been shown to be potential vectors of zoonotic diseases and, together with small rodents, play an important role in the distribution of viruses (hantavirus, Saint-Louis encephalitis virus, tick-borne encephalitis virus, etc.), bacteria (*Francisella tularensis*, *Salmonella typbimurium*, *Borrelia bugdorferi*, etc.) and protozoan infections (*Trypanosoma cruzi*, etc.) (Moro *et al*., 2005).

The genus *Eulaelaps* is zoologically classified under Acari, Parasitiformes, Mesostigmata, Gamasina and Haemogamasidae (Vinarski and Korallo-Vinarskaya, 2017). Most species of the genus *Eulaelaps* are blood-sucking parasites, found mainly in the nests and shelters of small mammals (Vinarski and Korallo-Vinarskaya, 2017). *Eulaelaps stabularis* is considered to be the most common mite found on the nests and bodies of small rodents and insectivores (Turk, 1945; Allred, 1969). Some mites of the genus *Eulaelaps* have morphological characters that are very similar, leading to a high degree of confusion in the identification of mites of the genus *Eulaelaps* by different scholars. For example, synonyms of *E. stabularis* include *Gamasus stabularis*, *E. arcualis*, *E. oribatoides*, *E. oudemansi*, *Laelaps propheticus*, etc. (Korneev, 2003; Vinarski and Korallo-Vinarskaya, 2017). Synonyms of *E. kolpakovae* include *E. novus* (Vinarski and Korallo-Vinarskaya, 2017). Some mites of the genus *Eulaelaps* carry a variety of zoonotic pathogens, e.g., *E. stabularis* and *E. shanghaiensis* can transmit hemorrhagic fever with renal syndrome (Moro *et al*., 2005; Huang *et al*., 2013), and *E. kolpakovae* carries *Yersinia pestis* (Sludsky, 2014).

The mitochondria are a semi-autonomous organelle found in almost all eukaryotes and are the main site of oxidative phosphorylation (Bi *et al*., 2020). The mitochondrial genome, with its simple structure, matrilineal inheritance, low recombination rate, and rapid evolution, has been widely used in studies of parasite classification, species identification, molecular evolution, phylogenetics and population genetics (Gao *et al*., 2022; Yang *et al*., 2022). The mitochondrial genome is usually composed of 37 genes, that is, 13 protein-coding genes (PCGs), 22 transporter RNA genes (tRNAs), 2 ribosomal RNA genes (rRNAs) and a non-coding region (control regions, CR) (Yang *et al*., 2023). In recent years, with the rapid development of genome assembly technologies and the gradual reduction of sequencing costs, the mitochondrial genomes of an increasing number of species have been sequenced. At the same time, the use of mitochondrial genomes to explore the phylogenetic relationships among species has become a hot topic of research (Cameron, 2014; Chen *et al*., 2022*b*).

Currently, for none of the species within the genus *Eulaelaps* mitochondrial genomes have been sequenced. In order to further fill the gap in understanding the molecular evolution of species within the genus *Eulaelaps*. Hence, in this study, the mitochondrial genome of *E. silvestris* parasitizing *Apodemus chevrieri* was sequenced and assembled, and a phylogenetic tree was constructed using molecular systematics-related methods. The results provide a valuable resource for further research on the genetic diversity and phylogenetic analysis of mites in the genus *Eulaelaps*.

## Materials and methods

### Sample collection and morphological identification

**Fig. 1.**
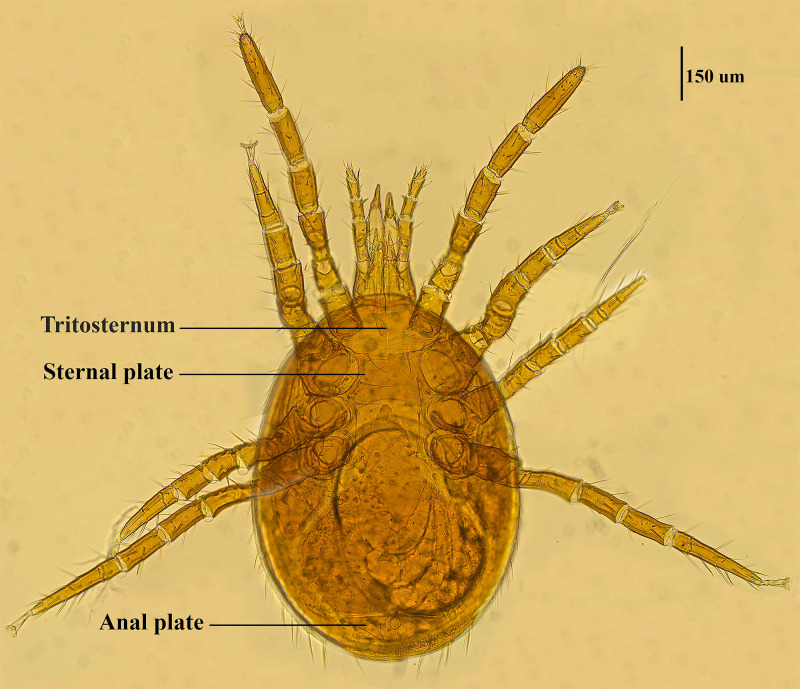
Morphological characteristics of *Eulaelaps silvestris*.

Mite specimens were collected from the body surface of *A. chevrieri* in Hongyuan County, Sichuan Province, China. Collected mite specimens were stored in EP tubes containing 95% ethanol, and the sample number was labeled 161. In the laboratory, collected mite specimens were directly placed under a SZ2-ILST dissecting microscope (Olympus, Tokyo, Japan) for species identification. One sample was taken and mounted on a glass slide with Hoyer's solution, and after dehydration, drying and transparency, the mounted sample was photographed under a Leica DM 3000 LED microscope (Lecia, Weztlar, Germany) to obtain pictures of the morphological characteristics of the sample. The mite was morphologically identified according to the identification basis in the references (Deng *et al*., 1993), and the mite was identified as *Eulaelaps silvestris* (Fig. 1). *Eulaelaps silvestris*’ main distinguishing characteristics are: tritosternum is smaller. The sternal plate anteriorly presents a transversely flattened reticular area with a dense reticulation of inverted teeth. The sternal plate is concave on both the anterior and posterior edges. The genital plate has a deeper notch where it heals with the ventral plate but does not have grooves inward. The anal plate is flat and triangular, with a central bulge on the anterior edge.

### DNA extraction and mitochondrial genome sequencing

The adult mites were removed from EP tubes containing 95% ethanol and immersed in sterile distilled water for 30 minutes to remove other microorganisms from the surface, then the mites were cut with a sterile scalpel blade. Genomic DNA was extracted using the DNeasy Blood and Tissue Kit (Qiagen, Valencia, California, USA). The extracted DNA was used to construct Illumina PE libraries. The mitochondrial genome was then sequenced on the Illumina Novoseq 6000 platform (Winnerbio, Shanghai, China). The raw data were processed using Trimmomatic v. 0.35 (Bolger *et al*., 2014) to remove low-quality reads and obtain high-quality clean data. Finally, 4.47 GB of clean data were obtained for the assembly of the complete mitochondrial genome of *E. silvestris*.

### Mitochondrial genome annotation

The mitochondrial genome was assembled using MitoZ 2.3 (https://doi.org/10.1101/489955). The MITOS web server predicted genes (Bernt *et al*., 2013), and 13 PCGs were compared and edited using the BLAST tool from NCBI. The location and length of tRNA genes were further confirmed using tRNAscan-SE (Lowe and Eddy, 1997) and ARWEN (Laslett and Canbäck, 2008). The positions of the 2 rRNA genes were determined based on their putative secondary structures and previously sequenced mitochondrial genomes. The location of the CR was confirmed based on the boundaries of adjacent genes. The mitochondrial sequence of *E. silvestris* has been deposited in GenBank under the accession number OQ184757. The associated SRA is SRR24658662.

### Sequence analysis and phylogenetic reconstruction

Base composition and codon usage were calculated using Geneious v.2020.2 Prime (created by Biomatters; available from https://www.geneious.com) and MEGA X (Kumar *et al*., 1994), respectively. Calculation of AT-skew and GC-skew was performed according to the formulas [AT-skew = (A – T)/(A + T); GC-skew = (G – C)/(G + C)] (Perna and Kocher, 1995). The Tandem Repeats Finder Program (http://tandem.bu.edu/trf/trf.html) was used to predict tandem repeat sequences in the CR, and CodonW1.4.2 (https://sourceforge.net/projects/codonw/) was used to determine the relative synonymous codon usage (RSCU) of the 13 PCGs. *Limulus polyphemus* (JX983598) and *Carcinoscorpius rotundicaud* (MW446894) were selected as outgroups, and phylogenetic trees were constructed using MrBayes 3.2.7 (Huelsenbeck and Ronquist, 2001) and IQ-TREE (Nguyen *et al*., 2015) using the Bayesian inference (BI) method and maximum likelihood (ML) method, respectively. Sequence alignment was performed using MAFFT (Katoh *et al*., 2002). According to the Bayesian information criterion, ModelFinder (Kalyaanamoorthy *et al*., 2017) was used to determine the best nucleotide substitution model for constructing ML and BI trees (Supplementary materials 1 and 2). Bayesian trees were run for a total of 1 000 000 generations and sampling every 1000 generations; 4 independent Monte Carlo Markov chains were run simultaneously. The first 25% of the trees were burned to ensure sample independence. To estimate the support of the BI tree, the posterior probability (PP) was calculated. For the ML tree, 50 000 ultra-fast bootstrap replications were used to calculate branch reliability (bootstrap probability, BP). The constructed phylogenetic trees were viewed and edited using FigTree 1.4.4 (https://github.com/rambaut/figtree/). The species and accession numbers for the constructed phylogenetic tree are shown in Supplementary material 3.

## Results

### Mitochondrial genome characteristics of *E. silvestris*

**Fig. 2.**
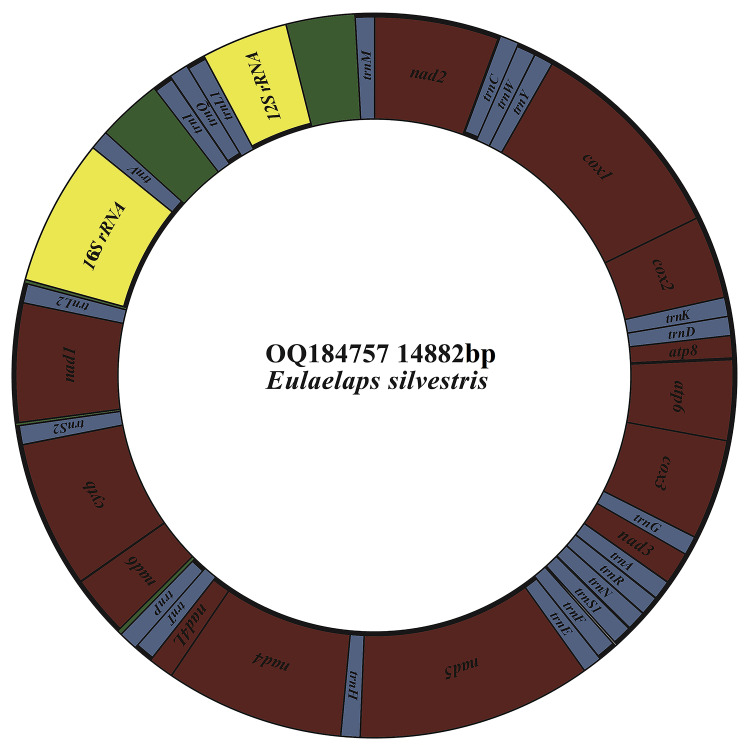
Mitochondrial genome loop map of *Eulaelaps silvestris*.

The mitochondrial genome of *E. silvestris* is a double-stranded DNA molecule with a length of 14 882 bp, consisting of 13 PCG genes (*cox1*-*3*, *nad1*-*6*, *nad4L*, *atp6*, *atp8*, *cytb*), 22 tRNA genes, 2 rRNA genes (*rrnS* and *rrnL*) and 2 CR (Fig. 2, Table 1). Of the 37 genes, 23 genes (*cox1-3*, *nad2-3*, *nad6*, *atp6*, *atp8*, *cytb*, *trnA*, *trnF*, *trnD*, *trnL1*, *trnG*, *trnI*, *trnK*, *trnM*, *trnN*, *trnR*, *trnS1*, *trnS2*, *trnT*, *trnW*) encode on the J-strand. The remaining 14 genes (*nad1*, *nad4*, *nad4L*, *nad5*, *trnC*, *trnH*, *trnY*, *trnE*, *trnP*, *trnL2*, *trnV*, *trnQ*, *16S rRNA* and *12S rRNA*) are encoded on the N-strand. The complete mitochondrial genome of *E. silvestris* consisted of 5244 adenine (A), 5007 thymine (T), 2943 cytosine (C) and 1688 guanine (G) in proportions of 35.2, 33.6, 19.8 and 11.4%, respectively (Table 2). The A + T (68.8%) content was much higher than the G + C (31.2%) content, with the lowest content of G. The AT-skew for the complete mitochondrial genome of *E. silvestris* was 0.02 and the GC-skew was −0.27. The AT content of each region showed a trend of tRNAs > CR > rRNAs > PCGs (Table 2).

**Table 1.** Organization of the *Eulaelaps silvestris* mitochondrial genome

Gene	Strand	Position	Length (bp)	Initiation codon	Termination codon	Anticodon	Intergenic nucleotide
*nad2*	J	2–958	957	ATT	TAA		1
*trnC*	N	951–1012	62			GCA	−8
*trnW*	J	1013–1074	62			UCA	0
*trnY*	N	1073–1133	61			GUA	−2
*cox1*	J	1135–2667	1533	ATC	TAA		1
*cox2*	J	2667–3338	672	ATG	TAA		−1
*trnK*	J	3339–3400	62			CUU	0
*trnD*	J	3401–3462	62			GUC	0
*atp8*	J	3463–3621	159	ATA	TAA		0
*atp6*	J	3612–4277	666	ATG	TAA		−10
*cox3*	J	4278–5066	789	ATA	TAA		
*trnG*	J	5066–5127	62			UCC	−1
*nad3*	J	5128–5463	336	ATT	TAG		
*trnA*	J	5462–5522	61			UGC	−2
*trnR*	J	5527–5580	54			UCG	4
*trnN*	J	5578–5643	66			GUU	−3
*trnS_1_*	J	5636–5688	53			GCU	−8
*trnF*	J	5694–5753	60			GAA	5
*trnE*	N	5752–5812	61			UUC	−2
*nad5*	N	5813–7487	1675	ATA	T		0
*trnH*	N	7488–7548	61			GUG	0
*nad4*	N	7549–8851	1303	ATG	T		
*nad4L*	N	8853–9125	273	ATT	TAG		1
*trnT*	J	9127–9192	66			UGU	1
*trnP*	N	9193–9254	62			UGG	0
*nad6*	J	9286–9720	435	ATT	TAA		31
*cytb*	J	9717–10 823	1107	ATA	TAG		−4
*trnS_2_*	J	10 823–10 875	53			UGA	−1
*nad1*	N	10 896–11 807	912	ATT	TAG		20
*trnL_2_*	N	11 808–11 868	61			UAA	0
*16S rRNA*	N	11 892–12 984	1093				23
*trnV*	N	12 985–13 047	63			ACU	0
*CR1*		13 048–13 478	431				0
*trnI*	J	13 479–13 542	64			GAU	0
*trnQ*	N	13 540–13 605	66			UUG	−3
*trnL_1_*	J	13 607–13 669	63			UAG	1
*12S rRNA*	N	13 670–14 362	693				0
*CR2*		14 363–14 819	457				0
*trnM*	J	14 820–14 882	63			CAU	0

**Table 2.** Base composition in the *Eulaelaps silvestris* mitochondrial genome

*silvestris*	Size (bp)	A%	T%	G%	C%	AT%	GC%	AT-skew	GC-skew
Mitogenome	14 882	35.2	33.6	11.4	19.8	68.8	31.2	0.02	−0.27
PCGs	10 817	35.1	32.2	11.8	20.9	67.3	32.7	0.04	−0.28
tRNAs	1348	38.0	35.8	10.6	15.6	73.8	26.2	0.03	−0.19
rRNAs	1786	34.0	38.7	8.30	19.0	72.7	27.3	−0.06	−0.39
CR	888	36.0	37.6	13.3	13.1	73.6	26.4	−0.01	0.02
*cox1*	1533	29.0	33.1	15.1	22.8	62.1	37.9	−0.07	−0.20
*cox2*	672	31.8	34.5	12.5	21.2	66.3	33.7	−0.04	−0.26
*cox3*	789	26.2	35.2	16.0	22.6	61.4	38.6	−0.15	−0.17
*nad1*	912	41.3	26.5	11.4	20.7	67.8	32.1	0.22	−0.29
*nad2*	957	32.1	39.3	10.3	18.3	71.4	28.6	−0.10	−0.28
*nad3*	336	29.5	40.2	10.4	19.9	69.7	30.3	−0.15	−0.31
*nad4*	1303	45.4	26.4	11.0	17.2	71.8	28.2	0.26	−0.22
*nad4L*	273	47.3	23.7	7.0	22.0	71.0	29.0	0.33	−0.52
*nad5*	1675	43.6	26.4	10.4	19.6	70.0	30.0	0.25	−0.31
*nad6*	435	30.3	39.5	8.5	21.7	69.8	30.2	−0.13	−0.44
*atp6*	666	29.0	38.2	10.4	22.4	67.2	32.8	−0.14	−0.37
*atp8*	159	37.1	32.1	6.9	23.9	69.2	30.8	0.07	−0.55
*cytb*	1107	28.5	34.5	12.9	24.1	63.0	37.0	−0.10	−0.30
*rrnL*	1093	34.6	38.7	7.6	19.1	73.3	26.7	−0.06	−0.43
*rrnS*	693	33.0	38.8	9.4	18.8	71.8	28.2	−0.08	−0.33

The complete mitochondrial genome of *E. silvestris* contains 10 gene inter-genic regions and 12 gene overlap regions (Table 1), with a total length of 91 bp (ranging from 1 to 31 bp) and 45 bp (ranging from 1 to 10 bp), respectively. Intergenic regions were present at 20 gene junctions. The longest intergenic sequence (31 bp) occurs between *trnP* and *nad6*; the shortest intergenic sequence (1 bp) occurs between *trnY* and *cox1*, *nad4* and *nad4L*, *nad4L* and *trnT*, *trnQ* and *trnL_1_*, *trnM* and *nad2*. Overlapping regions were present at 24 gene junctions. The longest overlapping sequence (10 bp) occurs between *atp8* and *atp6*; the shortest overlapping sequence (1 bp) occurs between *cox1* and *cox2*, *cox3* and *trnG*, *cytb* and *trnS_2_* (Table 1). The other 26 gene junctions were without gene intragenic and overlapping regions.

### Protein-coding genes and relative synonymous codon usage

The 13 PCGs of *E. silvestris* had a total length of 10 817 bp, representing 72.7% of the length of the complete mitochondrial genome. AT content was 67.3%, similar to the AT content of the complete mitochondrial genome (Table 1). The shortest and longest genes were *atp8* (159 bp) and *nad5* (1,675 bp), respectively. All PCGs started with the typical ATN as an initiation codon (ATT: 5, ATA: 4, ATG: 3, ATC: 1), and no rare initiation codons (GTG and TTG) were present. In the use of termination codons, 11 PCGs (*cox1*-*3*, *nad1*, *nad2*, *nad3*, *nad4L*, *nad6*, *atp6*, *atp8*, *cytb*) ended with the complete termination codons TAA/TAG (TAA: 6, TAG: 4), and only 2 PCGs (*nad4* and *nad5*) had the incomplete termination codons T. The AT-skew of 13 PCGs was at -0.15 (*nad3*) to 0.33 (*nad4L*) and the GC-skew at –0.55 (*atp8*) to –0.17 (*cox3*). The AT content of individual protein genes was over 60%, and the highest AT content was found in *nad2*, *nad4* and *nad4L*, while the lowest AT content was found in *cox1* and *cox3* genes. Meanwhile, there were overlaps between PCGs, such as 1 bp between *cox1* and *cox2*, 4 bp between *nad6* and *cytb* genes, and 10 bp between *atp8* and *atp6* (Table 1).

**Fig. 3.**
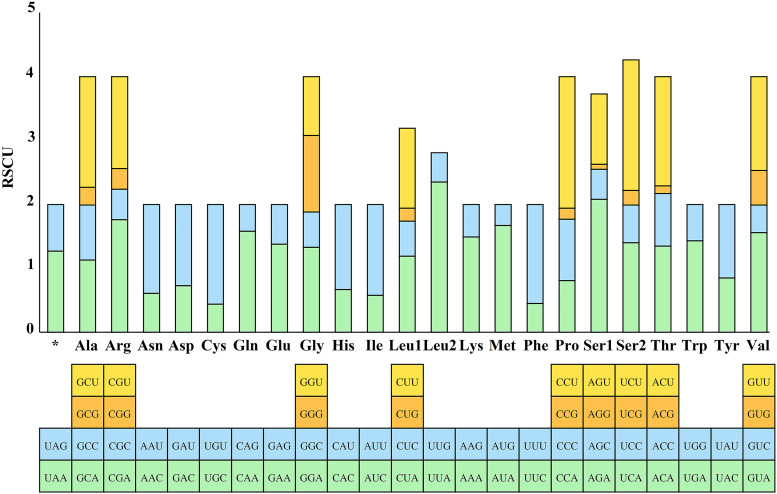
Relative synonymous codon usage (RSCU) of *Eulaelaps silvestris*. The *Y*-axis indicates the RSCU value, and the *X*-axis indicates the codons corresponding to the respective amino acids.

The RSCU values for the 13 PCGs of *E. silvestris* were further analysed and are shown in Fig. 3. The preference for relative synonymous codon usage is related to a number of factors, such as mutational pressure, gene length, gene function, natural selection, etc. (Behura and Severson, 2013; Chen *et al*., 2014). RSCU values indicate strong codon usage preference (RSCU > 1), no preference (RSCU = 1) and weak preference (RSCU < 1) (Gupta and Singh, 2021). In addition to the termination codons, the 13 PCGs contain a total of 3594 codons (Supplementary material 4), which are within the codon number of arthropods (3585–3746) (Cha *et al*., 2007). Phenylalanine (Phe), isoleucine (Ile) and leucine (Leu_1_) are the most common amino acids, while cysteine (Cys), arginine (Arg), glutamine (Gln) and aspartic acid (Asp) are the less common amino acids. From Fig. 3, the 5 most frequently used codons in the *E. silvestris* PCGs are UUA (Leu): 2.35, AGA (Ser): 2.08, CCU (Pro): 2.06, UCU (Ser): 2.04, CGA (Arg): 1.76, and all end in A/U. *Eulaelaps silvestris* mitochondrial PCGs with RSCU > 1 codons preferentially end in A/U; while codons with RSCU < 1 mostly end in G/C, with the exception of GGG (Gly), which has an RSCU of 1.20.

### Transfer RNA, ribosomal RNA genes and control regions

**Fig. 4.**
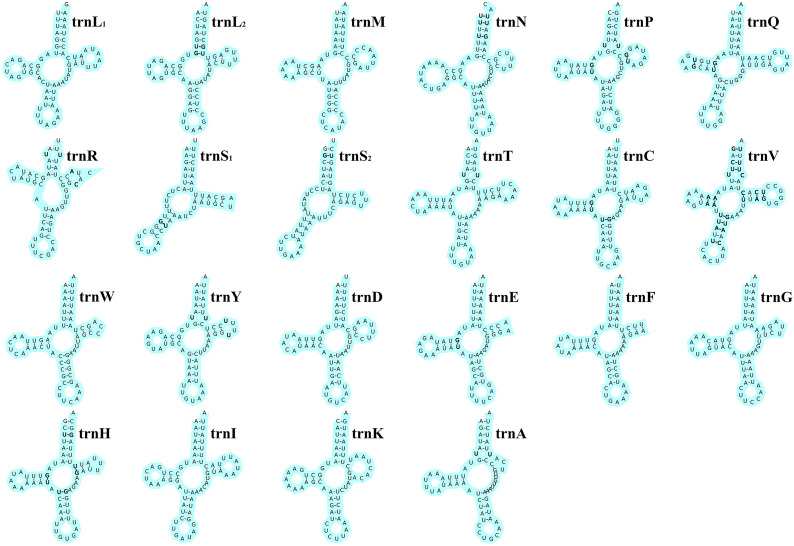
*Eulaelaps silvestris* tRNA gene putative secondary structure. Bold indicates mismatch.

The total length of the 22 tRNA genes in *E. silvestris* was 1348 bp, with a total AT content of 73.8%, a positive AT-skew (0.03) and a negative GC-skew (−0.19). The length of individual tRNA genes ranged from 53 bp (*trnS_1_* and *trnS_2_*) to 66 bp (*trnT*, *trnQ* and *trnN*), and the secondary structures of the 22 tRNA genes are shown in Fig. 4. All of the other 20 tRNA genes are capable of forming a typical cloverleaf secondary structure, except for *trnS_1_* and *trnS_2_*, which lack the D arm and cannot form a typical cloverleaf secondary structure. The anticodon arms of the 22 tRNA genes in *E. silvestris* consist of 4 (*trnL_1_* only) to 5 nucleotide pairs, the amino acid acceptor arms consist of 5 (*trnR* only) to 7 nucleotide pairs, and the anticodon loops are all composed of 7 nucleotides. In addition to the typical Watson–Crick pairings (A–U, G–C), a total of 38 base mismatches occurred in the *E. silvestris* mitochondrial genome for 22 tRNA genes during the folding process, including G–U mismatches (19), U–U mismatches (10), U–C mismatches (4), A–A mismatches (3) and A–C mismatches (2). Among them, *trnH* had the most G–U mismatches (4), *trnN*, *trnV* and *trnY* had the most U–U mismatches (2), *trnV* had the most U–C mismatches (4), *trnV* had the most A–A mismatches (3) and *trnR* and *trnV* had 1 A–C mismatch each.

*Eulaelaps silvestris*’ 2 rRNA genes (*16S rRNA* and *12S rRNA*) are 1096 bp and 693 bp in size, respectively (Table 1). The *16S rRNA* and *12S rRNA* are encoded by the N-strand, same as the 2 rRNA genes of most Acari and arthropods. The *16S rRNA* gene was located between *trnL_2_* and *trnV* with 73.3% AT content, while the *12S rRNA* gene was located between *trnL_1_* and CR2 with 71.8% AT content, which was slightly lower than the AT content of the *16S rRNA* gene. Duplicated control regions (CR1 and CR2) were identified in the *E. silvestris* mitochondrial genome. CR1 and CR2 are 431 and 457 bp in length, respectively, and share a common identical core sequence of 308 bp (positions 13 135–13 442 for CR1 and 14 468–14 775 for CR2). CR1 is located between *trnV* and *trnI*, and CR2 is located between *rrnS* and *trnM*. In the first CR, 2.4 tandem repeats were present, and no tandem repeats were found in the second CR.

### Gene rearrangement and phylogenetic analysis

**Fig. 5.**
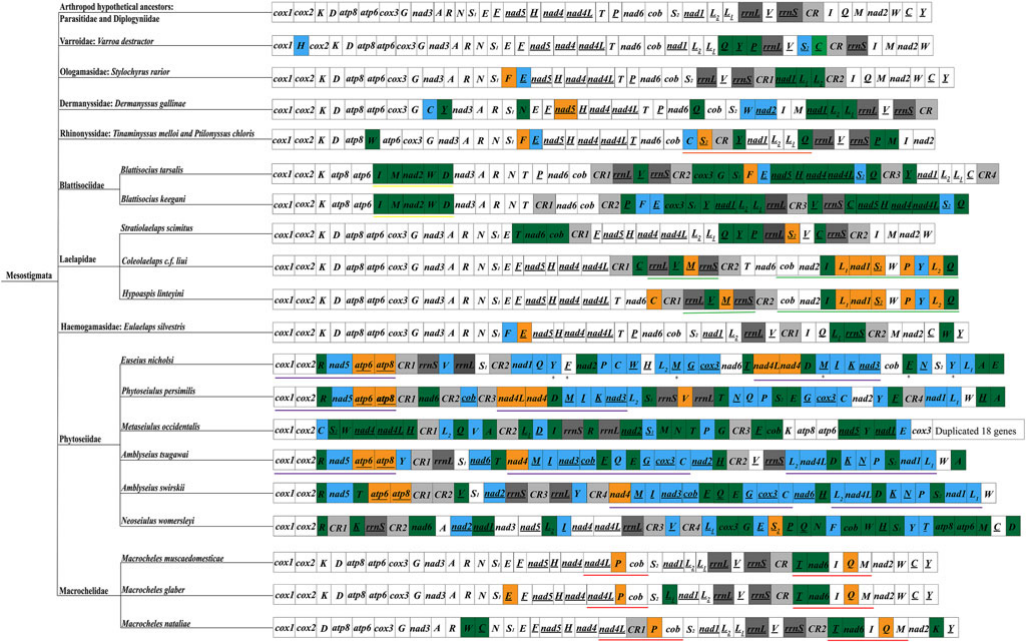
Mitochondrial genome arrangement pattern of Mesostigmata. Black underline indicates that the gene is located on the N-strand; blue indicates that the gene is inverted and translocated; green indicates that the gene is translocated; orange indicates that the gene is inverted. Two rRNA genes are indicated in dark grey; control region is indicated in light grey. ✱ indicates a duplicated gene; same colour underlines indicate shared gene clusters.

The arrangement order of the mitochondrial genome reflects, to some extent, information on the molecular evolution of mitochondria and is potentially valuable in resolving certain controversial phylogenetic relationships (Tan *et al*., 2018; Zhang *et al*., 2020). The rearrangement patterns of Mesostigmata sequenced species were analysed using the mitochondrial genome arrangement patterns of hypothetical arthropod ancestors (*L. polyphemus*) as a reference (Staton *et al*., 1997) (Fig. 5). Among Mesostigmata, only species in the families Parasitidae and Diplogyniidae had the same mitochondrial genome arrangement pattern as that of hypothetical arthropod ancestors, while in the remaining 9 families (Varroidae, Ologamasidae, Dermanyssidae, Laelapidae, Haemogamasidae, Blattisociidae, Rhinonyssidae, Macrochelidae and Phytoseiidae), 20 species of mites were rearranged to different degrees. More importantly, 5 families (Rhinonyssidae, Blattisociidae, Laelapidae, Macrochelidae and Phytoseiidae) were also found to share the same gene clusters among species within families or genera, i.e. Rhinonyssidae intra-family species share 1 gene cluster (*trnC*-*trnS_2_*-*trnY*-*nad1*-*trnL_2_*-*trnL_1_*-*trnQ*) (note: underlined genes and non-underlined genes indicate that the genes are located on different DNA strands); species of the genera *Colaelaps* and *Hypoaspis* within the family Laelapidae share 2 gene clusters (*rrnL*-*trnV*-*trnM-rrnS* and *cob*-*nad2*-*trnI*-*trnL_1_*-*nad1*-*trnS_2_*-*trnW*-*trnP*-*trnY*-*trnL_2_*-*trnQ*); species within the genus *Blattisocius* in the family Blattisociidae share 1 gene (*trnI*-*trnM*-*nad2*-*trnW*-*trnD*); species within the genus *Macrocheles* in the family Macrochelidae share 2 gene clusters (*nad4L*-*trnP*-*cob* and *trnT*-*nad6*-*trnI*-*trnQ*-*trnM*); 3 species in different genera of the family Phytoseiidae (*Phytoseiulus persimilis*, *Euseius nicholsi* and *Amblyseius tsugawai*) share 1 gene cluster (*cox1*-*cox2*-*trnR*-*nad5*-*atp6*-*atp8*), and interestingly, *P. persimilis* and *E. nicholsi* also share 1 gene cluster (*nad4L*-*nad4*-*trnD*-*trnM*-*trnI*-*trnK*-*nad3*) and there are 2 additional gene clusters (*nad4*-*trnM*-*trnI*-*nad3*-*cob*-*trnF*-*trnQ*-*trnE*-*trnG*-*cox3*-*trnC* and *trnL_2_*-*nad4L*-*trnD*-*trnK*-*trnN*-*trnP*-*trnS_2_*-*nad1*-*trnL_1_*) shared by species of the genus *Amblyseius*. In this study, *E. silvestris* is a new rearrangement pattern in Mesostigmata and does not share gene clusters with other families. The rearrangements in *E. silvestris* occurred mainly in 3 regions, that is, the 3 genes *trnL_1_*, *rrnS* and *trnW* underwent translocations; the *trnF* gene underwent inversions; the *trnE* gene underwent translocations and inversions.

**Fig. 6.**
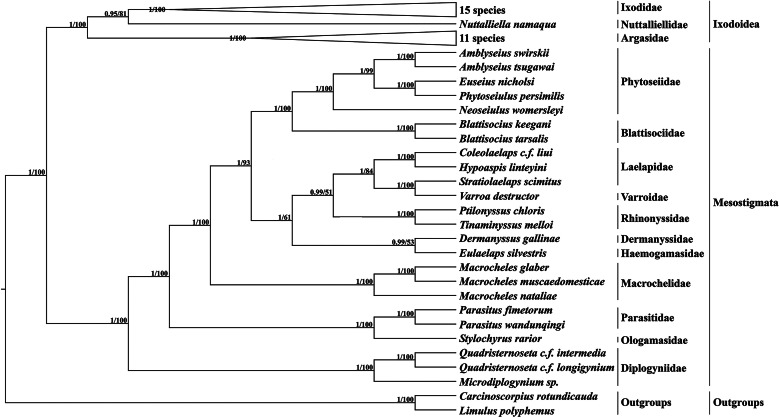
Phylogenetic tree constructed based on 13 protein-coding genes. The numbers beside the nodes are posterior probabilities (BI) and bootstrap (ML), respectively.

Based on 13 PCGs with *L. polyphemus* and *C. rotundicauda* as outgroups, a phylogenetic tree was constructed using the ML method and the BI method (Fig. 6). Both methods obtained a phylogenetic tree with identical topology but different node support. Most branches of the BI tree generally had greater support than the ML tree, and 3 nodes of the ML tree were less than 70. According to the phylogenetic tree results, Mesostigmata is a monophyletic group, and species of the family Diplogyniidae are the early differentiated taxa in Mesostigmata. Most of the mites of the same taxonomic order cluster separately into 1 group, except for the family Laelapidae. In the present study, *E. silvestris* and species of the family Dermanyssidae clustered together to form a sister branch.

## Discussion

In this study, the complete mitochondrial genome of *E. silvestris* was sequenced and analysed. The mitochondrial genome of *E. silvestris*, like that of other mites, is a double-stranded DNA molecule containing 37 genes, with a total length of 14 885 bp within the range of the mitochondrial genomes in Mesostigmata sequenced species (Li *et al*., 2019). It is thought that the main cause of differences in mitochondrial genome size is the number and length of CR, which are positively correlated with species’ mitochondrial genome size, and that differences in the number and length of CR can occur between species (Zhang and Hewitt, 1997). Of the reported mitochondrial genomes of Mesostigmata mites so far, *Metaseiulus occidentalis* has the largest mitochondrial genome (24 961 bp), but does not possess the greatest number or length of CR; in contrast, *Blattisocius tarsalis* (16 454 bp) has the greatest number and length of CR, but not the largest mitochondrial genome; *Quadristernoseta c. f. intermedia* has the smallest mitochondrial genome (14 423 bp), and the CR was not the shortest in length (Li *et al*., 2019). It can be inferred that the mitochondrial genome sequence length differences in Mesostigmata mites are not significantly correlated with the number and length of CR. Tandem repeats are only present in the first CR of *E. silvestris*. Tandem repeats have been found only in the CR of the *Varroa destructor* mitochondrial genome (Navajas *et al*., 2002). It is speculated that tandem repeats may be associated with individual differences in species or life activity levels.

The AT content in the complete mitochondrial genome of *E. silvestris* was 68.8%, which was lower than the average A + T content of the Acari mitochondrial genome (75.34 ± 4.78%) (Yuan *et al*., 2010). The regions with the highest AT content are tRNA genes, and the regions with the lowest AT content are PCGs. It is generally believed that the low AT content of PCGs facilitates the function of encoding the required proteins (Monroe *et al*., 2022). The strand skew of the total nucleotide composition, PCGs and tRAN genes was the same as that of metazoa (positive for AT-skew and negative for GC-skew) (Hassanin *et al*., 2005), while the rRNA genes had negative values for both AT-skew and GC-skew, indicating that the total nucleotide composition, PCGs and tRAN genes were AT-skewed and the rRNA genes were TA-skewed. The nucleotide composition strand bias is associated with asymmetric mutational and selective pressures on the 4 bases during replication and transcription (Hassanin *et al*., 2005). Thus, the nucleotide composition preference in the *E. silvestris* mitochondrial genome is an important reference for subsequent studies on the replication and transcriptional mechanisms of mitochondrial genomes in species of the family Haemogamasidae.

One of the notable characteristics of the metazoan mitochondrial genome is its more compact intergenic structure. The *E. silvestris* mitochondrial genome has a total of 10 intergenic regions. The presence of intergenic genes generally promotes organismal evolution because intergenic genes facilitate the storage of more genetic information and increase the probability of mutation. Boyce *et al*. (1989) suggested that the overlap phenomenon occurring in the mitochondrial genome facilitates the miniaturization of the mitochondrial genome, and that miniaturized mitochondrial genomes have an advantage in natural selection because the time required for their replication is greatly reduced. The *E. silvestris* mitochondrial genome has a total of 12 gene overlap regions that are shorter than the length of the gene intergenic regions. The gene intergenic regions and gene overlap regions that occur in the mitochondrial genome increase the stability of the mitochondrial structure. Some of the metazoan 13 PCGs exist overlapping each other; for example, the *nad4* and *nad4L*, *atp8* and *atp6* genes are often found to have an overlap of about 7 bp at their neighboring junctions in the mitochondrial genomes of some insects and ticks (Black and Roehrdanz, 1998; Li *et al*., 2023). In this study, the 13 PCG junctions of *E. silvestris* only overlap by 10 and 4 bp between *atp8* and *atp6* and between *nad6* and *cytb*, respectively. The 10 bp overlap between the *atp8* and *atp6* genes has been widely reported in the mitochondrial genomes of some bird species (Desjardins and Morais, 1991; Nishibori *et al*., 2002).

Compared to the nuclear genome, animal mitochondrial genomes have their own characteristics for the use of codons in the 13 PCGs (Liu and Schultz, 2010), with most genes starting with ATN and some rare initiation codons, such as TTG (Zhang *et al*., 2022), GTG (Pavan-Kumar *et al*., 2022), CGA (Chen *et al*., 2022*a*), etc. *Eulaelaps silvestris*’ 13 PCGs were all initiated with a typical ATN, and no rare initiation codons were present. Only 2 PCGs (*nad4* and *nad5*) ended with incomplete termination codons T, whereas the other 11 PCGs had complete termination codons. Incomplete termination codons are common in animal mitochondrial genomes, and it has been demonstrated that these incomplete termination codons can be modified into complete termination codons by polyadenylation of the 3′ end during mRNA processing (Huang *et al*., 2022). Among the 13 PCG codons used, codons ending in A/U were more frequent than those ending in G/C, which may be one of the important reasons for the distinct AT preference in the *E. silvestris* mitochondrial genome base composition.

Metazoa mitochondrial genome tRNA gene is a cloverleaf secondary structure containing 4 arms and 4 loops, namely amino acid acceptor arm (AA-arm), dihydrouracil arm (DHU) and loop (D-arm and D-loop), anticodon arm and loop (AC-arm and AC-loop), pseudouracil arm and loop (TΨC-arm and TΨC-loop) and a variable loop (Zhang *et al*., 2019). Almost all animals *trnS_1_* lack the D arm, which is thought to be an ancestral characteristic of metazoa (Xue *et al*., 2018). In the 22 tRNA genes of *E. silvestris*, besides *trnS_1_*, which lacks the D arm, there is *trnS_2_* which has an atypical cloverleaf secondary structure (missing the D arm). The length of individual tRNA genes of *E. silvestris* ranged from 53 bp (*trnS1* and *trnS_2_*) to 66 bp (*trnT*, *trnQ* and *trnN*), with some tRNA genes within the average length (62.0 ± 1.3 bp) of tRNA genes of Parasitiformes species (Yuan *et al*., 2010). *Eulaelaps silvestris* 22 tRNA genes showed a total of 38 times of irregular pairing during the folding process. Among them, G–U mismatches occurred most frequently. It has been demonstrated that the base mismatches occurring in tRNA genes can be corrected by RNA editing without affecting the normal amino acid translocation function (Yokobori and Pääbo, 1995; Lavrov *et al*., 2000).

The mitochondrial genome contains 37 genes, which can be arranged in various orders and alignments, and can be used to infer the degree of evolution and relatedness of species by having the same gene order and alignment (Boore and Brown, 1994). In Mesostigmata, species from only 2 families (families Parasitidae and Diplogyniidae) share the same pattern of mitochondrial genome arrangement as the hypothetical arthropod ancestors, while the mitochondrial genomes of the remaining 9 families and 20 species have been rearranged to different degrees, overturning the previous conclusion that the mitochondrial genome arrangement and structure are abnormally stable. Some of the species in the same family or genus have the same gene clusters, suggesting that these identical gene clusters are derivations common to these families or genera. In the *E. silvestris* mitochondrial genome, only 5 genes have undergone rearrangements, and the rearrangements occurred mainly near tRNA genes and CR. It has been confirmed that the vicinity of tRNA genes and CR are hotspot regions where rearrangements occur, as replication of CR may lead to rearrangements (Kurabayashi *et al*., 2008; Yang *et al*., 2023). Thus, these rearranged genes or regions may become hotspots for mitochondrial genome research in the future.

The family Haemogamasidae is sometimes considered as a subfamily of the family Laelapidae, but this view has not been shared by most scholars (Vinarski and Korallo-Vinarskaya, 2017). Therefore, in this study, based on 13 PCGs, ML and BI trees were constructed, and both phylogenetic trees formed consistent topologies but had slightly different node supports. Both phylogenetic trees support Mesostigmata as a monophyletic group, consistent with the results of Li *et al*. (2019), but inconsistent with the conclusion of Lindquist *et al*. (2009*b*) that Mesostigmata is a polyphyletic group. With the exception of the family Laelapidae, the remaining related species clustered well together according to family order, indicating that the mesostigmata phylogenetic relationships are clear for most species within the family. In this study, *E. silvestris* formed a sister branch with species from the family Dermanyssidae, indicating that the family Haemogamasidae has the closest affinity to the family Dermanyssidae in Mesostigmata. Meanwhile, the phylogenetic analysis provided strong molecular corroboration that the family Haemogamasidae does not belong to the subfamily Laelapidae.

## Conclusion

In this study, the mitochondrial genome of *E. silvestris*, which is parasitic on the body surface of *A. chevrieri*, was sequenced and assembled, filling a gap in our understanding of the molecular evolution of species in the genus *Eulaelaps*. The results showed that *E. silvestris* is a double-stranded DNA molecule with a size of 14 882 bp. The intergenic arrangement is relatively compact, and the base composition shows a distinct AT preference. The mitochondrial genome of *E. silvestris* underwent a smaller rearrangement compared to the mitochondrial genome arrangement pattern of arthropod hypothetical ancestors. Phylogenetic analysis showed that the family Haemogamasidae is most closely related to the family Dermanyssidae. The results accumulate molecular data for the genus *Eulaelaps* and provide a theoretical basis for further studies on the phylogenetic relationships of the genus *Eulaelaps*.

## Data Availability

The complete mitochondrial genome sequence of *Eulaelaps silvestris* is available at the National Center for Biotechnology Information (NCBI) at [https://www.ncbi.nlm.nih.gov/] under accession number OQ184757. The associated SRA is SRR24658662.

## Supplementary material

For supplementary material accompanying this paper visit https://doi.org/10.1017/S0031182023000616. click here to view supplementary material
