# Supplementary material for: Description and phylogenetic analysis of the complete mitochondrial genome in Eulaelaps silvestris provides new insights into the molecular classification of the family Haemogamasidae
Source: Parasitology. 2023 Jul 3;150(9):821–30. doi: 10.1017/S0031182023000616 (PMC10478059; doi:10.1017/S0031182023000616)
Supplement: Supplementary file 1 [file S0031182023000616sup001.zip › S0031182023000616sup001.docx]

**Supplementary material 1.** ModelFinder is used to determine the best partitioning scheme and the corresponding best alternative model for each dataset for constructing the maximum likelihood tree and applying it to different analyses.

| Subset partitions | Best model |
| --- | --- |
| *atp6* codon1, *atp8* codon2, *nad3* codon1 | GTR+F+I+G4 |
| *atp6* codon2, *cox2* codon2, *cox3* codon2, *cytb* codon2, *nad3* codon2 | GTR+F+I+G4 |
| *atp6* codon3, *cox2* codon3, *cytb* codon3, *nad3* codon3 | GTR+F+G4 |
| *atp8* codon1, *nad2* codon1 | HKY+F+I+G4 |
| *atp8* codon3 | HKY+F+I+G4 |
| *cox1* codon1 | GTR+F+I+G4 |
| *cox1* codon2 | GTR+F+I+G4 |
| *cox1* codon3, *cox3* codon3 | GTR+F+I+G4 |
| *cox2* codon1, *cox3* codon1, *cytb* codon1 | GTR+F+I+G4 |
| *nad1* codon1, *nad4L* codon1, *nad4* codon1, *nad5* codon1 | GTR+F+I+G4 |
| *nad1* codon2, *nad4L* codon2, *nad4* codon2, *nad5* codon2 | GTR+F+I+G4 |
| *nad1* codon3 | HKY+F+ASC+G4 |
| *nad2* codon2, *nad6* codon2 | GTR+F+I+G4 |
| *nad2* codon3, *nad6* codon1, *nad6* codon3 | HKY+F+I+G4 |
| *nad4L* codon3 | HKY+F+ASC+G4 |
| *nad4* codon3, *nad5* codon3 | HKY+F+G4 |
