# Supplementary material for: Description and phylogenetic analysis of the complete mitochondrial genome in Eulaelaps silvestris provides new insights into the molecular classification of the family Haemogamasidae
Source: Parasitology. 2023 Jul 3;150(9):821–30. doi: 10.1017/S0031182023000616 (PMC10478059; doi:10.1017/S0031182023000616)
Supplement: Supplementary file 1 [file S0031182023000616sup001.zip › S0031182023000616sup003.docx]

**Supplementary material 3.** This study was used to construct a phylogenetic tree of species information.

| Family | Species | Accession number |
| --- | --- | --- |
| Varroidae | *Varroa destructor* | AJ493124 |
| Ologamasidae | *Stylochyrus rarior* | GQ927176 |
| Parasitidae | *Parasitus fimetorum* | NC061975 |
|  | *Parasitus wangdunqingi* | MK270528 |
| Dermanyssidae | *Dermanyssus gallinae* | MW044618 |
| Rhinonyssidae | *Tinaminyssus melloi* | MN557820 |
|  | *Ptilonyssus chloris* | MN557819 |
| Blattisociidae | *Blattisocius tarsalis* | MK270529 |
|  | *Blattisocius keegani* | MH120211 |
| Laelapidae | *Coleolaelaps c.f. liui* | MK270524 |
|  | *Hypoaspis linteyini*  *Stratiolaelaps scimitus* | MK270530  MK912042 |
| Phytoseiidae | *Phytoseiulus persimilis* | GQ222414 |
|  | *Euseius nicholsi* | KM999989 |
|  | *Amblyseius tsugawai* | MW729376 |
|  | *Amblyseius swirskii* | MW729377 |
|  | *Neoseiulus womersleyi* | MW762685 |
| Diplogyniidae | *Quadristernoseta c.f. intermedia* | MK270521 |
|  | *Quadristernoseta c.f. longigynium* | MK270522 |
|  | *Microdiplogynium sp.* | MK270523 |
| Macrochelidae | *Macrocheles glaber* | MK270525 |
|  | *Macrocheles muscaedomesticae* | MK270526 |
|  | *Macrocheles nataliae* | MK270527 |
| Haemogamasidae | *Eulaelaps silvestris* | OQ184757 |
| Ixodidae | *Ixodes cornuatus* | NC062630 |
|  | *Ixodes myrmecobii* | NC062632 |
|  | *Ixodes holocyclus* | NC005293 |
|  | *Ixodes hirsti* | NC062631 |
|  | *Ixodes australiensis* | NC062625 |
|  | *Ixodes uriae* | NC006078 |
|  | *Ixodes fecialis* | NC062628 |
|  | *Ixodes granulatus* | OL800705 |
|  | *Ixodes nipponensis* | NC058242 |
|  | *Ixodes persulcatus* | NC004370 |
|  | *Ixodes ricinus* | JN248424 |
|  | *Ixodes simplex* | KY457531 |
|  | *Ixodes tasmani* | NC041086 |
|  | *Ixodes vespertilionis* | MW411447 |
|  | *Ixodes woyliei* | NC062627 |
| Argasidae | *Antricola mexicanus* | KC769591 |
|  | *Ornithodoros moubata* | AB073679 |
|  | *Argas miniatus* | KC769590 |
|  | *Argas sp.* | KC769588 |
|  | *Ornithodoros brasiliensis* | KC769593 |
|  | *Ornithodoros rostratus* | KC769592 |
|  | *Otobius megnini* | KC769589 |
|  | *Ornithodoros porcinus* | AB105451 |
|  | *Argas lagenoplastis* | KC769587 |
|  | *Argas africolumbae* | JQ665720 |
|  | *Carios capensis* | AB075953 |
| Nuttalliellidae | *Nuttalliella namaqua* | NC019663 |
| Limulidae | *Limulus polyphemus* | JX983598 |
|  | *Carcinoscorpius rotundicauda* | JX437074 |
