# Supplementary figures and images for: Description and phylogenetic analysis of the complete mitochondrial genome in Eulaelaps silvestris provides new insights into the molecular classification of the family Haemogamasidae
Source: Parasitology. 2023 Jul 3;150(9):821–30. doi: 10.1017/S0031182023000616 (PMC10478059; doi:10.1017/S0031182023000616)

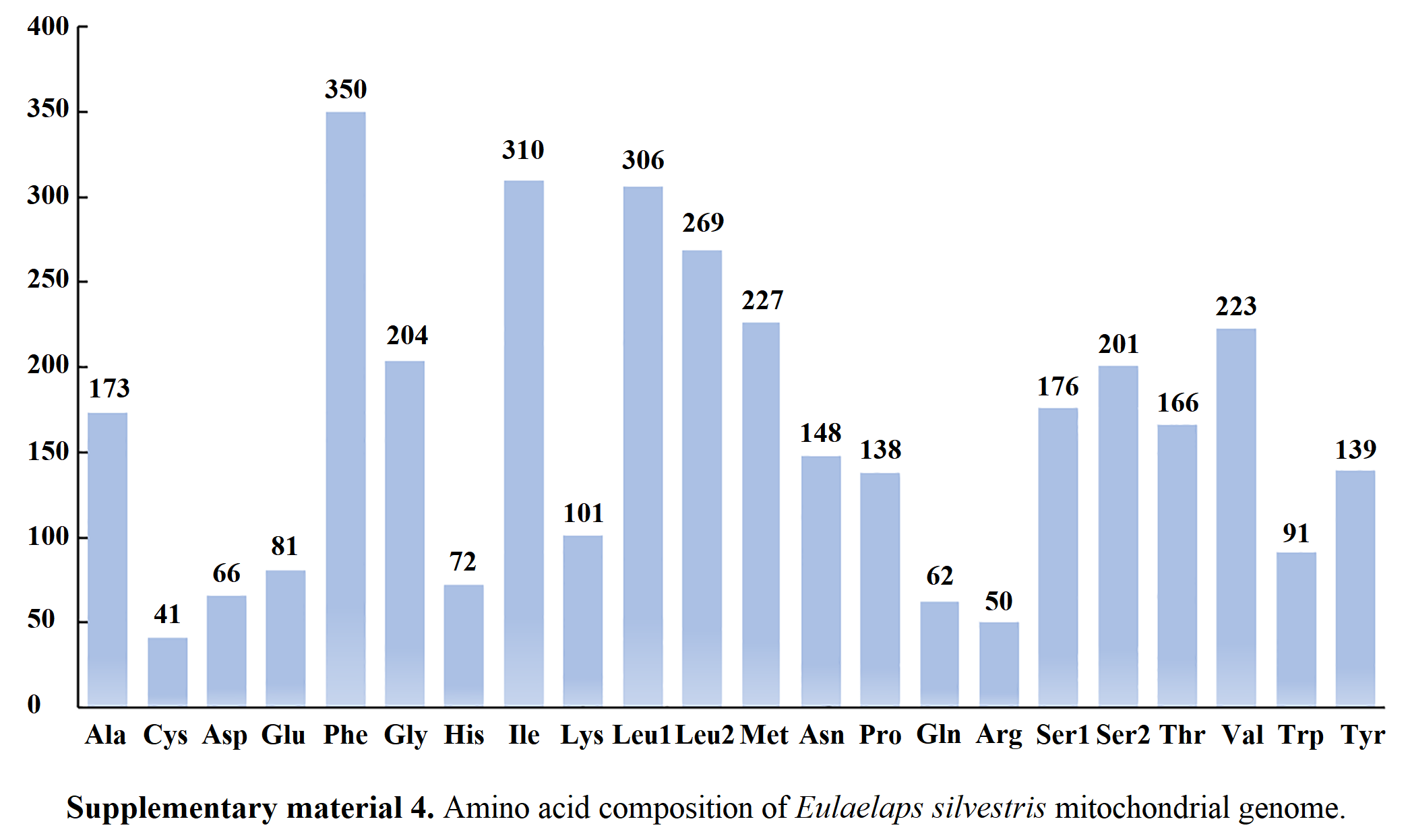

Supplement: Supplementary file 1 [file S0031182023000616sup001.zip › S0031182023000616sup004.tif]
